# Genetic architecture of atherosclerosis dissected by QTL analyses in three F2 intercrosses of apolipoprotein E-null mice on C57BL6/J, DBA/2J and 129S6/SvEvTac backgrounds

**DOI:** 10.1371/journal.pone.0182882

**Published:** 2017-08-24

**Authors:** Natalia Makhanova, Andrew P. Morgan, Yukako Kayashima, Andrei Makhanov, Sylvia Hiller, Svetlana Zhilicheva, Longquan Xu, Fernando Pardo-Manuel de Villena, Nobuyo Maeda

**Affiliations:** 1 Department of Pathology and Laboratory Medicine, University of North Carolina at Chapel Hill, Chapel Hill, United States of America; 2 Department of Genetics and the Lineberger Comprehensive Cancer Center, University of North Carolina at Chapel Hill, Chapel Hill, United States of America; 3 College of Computing, Georgia Institute of Technology, Atlanta, United States of America; Nagoya University, JAPAN

## Abstract

Quantitative trait locus (QTL) analyses of intercross populations between widely used mouse inbred strains provide a powerful approach for uncovering genetic factors that influence susceptibility to atherosclerosis. Epistatic interactions are common in complex phenotypes and depend on genetic backgrounds. To dissect genetic architecture of atherosclerosis, we analyzed F2 progeny from a cross between apolipoprotein E-null mice on DBA/2J (DBA-apoE) and C57BL/6J (B6-apoE) genetic backgrounds and compared the results with those from two previous F2 crosses of apolipoprotein E-null mice on 129S6/SvEvTac (129-apoE) and DBA-apoE backgrounds, and B6-apoE and 129-apoE backgrounds. In these round-robin crosses, in which each parental strain was crossed with two others, large-effect QTLs are expected to be detectable at least in two crosses. On the other hand, observation of QTLs in one cross only may indicate epistasis and/or absence of statistical power. For atherosclerosis at the aortic arch, *Aath4* on chromosome (Chr)2:66 cM follows the first pattern, with significant QTL peaks in (DBAx129)F2 and (B6xDBA)F2 mice but not in (B6x129)F2 mice. We conclude that genetic variants unique to DBA/2J at *Aath4* confer susceptibility to atherosclerosis at the aortic arch. A similar pattern was observed for *Aath5* on chr10:35 cM, verifying that the variants unique to DBA/2J at this locus protect against arch plaque development. However, multiple loci, including *Aath1* (Chr1:49 cM), and *Aath2* (Chr1:70 cM) follow the second type of pattern, showing significant peaks in only one of the three crosses (B6-apoE x 129-apoE). As for atherosclerosis at aortic root, the majority of QTLs, including *Ath29* (Chr9:33 cM), *Ath44* (Chr1:68 cM) and *Ath45* (Chr2:83 cM), was also inconsistent, being significant in only one of the three crosses. Only the QTL on Chr7:37 cM was consistently suggestive in two of the three crosses. Thus QTL analysis of round-robin crosses revealed the genetic architecture of atherosclerosis.

## Introduction

Atherosclerosis is the major cause of morbidity and mortality in North America. It is an inflammatory disease of arteries, characterized by the development of lipid-laden plaques affecting multiple vascular beds. Genetic factors play a crucial role in the pathogenesis of atherosclerosis, and genome-wide association studies (GWAS) in humans have yielded multiple loci and insights into their relevance to the disease [1]. However, the precise role of these loci is not fully understood, with the exception of a few genes such as *Ldlr* (low-density lipoprotein receptor) and *Apoe* (apolipoprotein E). This is because atherogenesis is a highly complex process on which each gene often has only small effects, and certain lifestyle characteristics, such as smoking, diet, and stress, have an impact on the development of this disease. Genetic analysis in animal models is a valuable tool to complement human GWAS since environmental factors can be carefully controlled. A large number of genetic loci affecting plaque development have yielded results utilizing hyperlipidemic *Apoe*-null mice, *Ldlr*-null mice or atherogenic diet-fed wild type mice [2], although defining causative variants/gene(s) within any QTL regions remains a challenge.

*Apoe*-null mice on different strain backgrounds show differences in susceptibility to atherosclerosis in a vascular location-specific manner [3]. Thus, *Apoe*-null mice on a C57BL/6J (B6) background (B6-apoE), develop atherosclerotic plaques initially at their aortic root and later, as they age, at other locations, including in the innercurve of aortic arch. In contrast, *Apoe*-null mice on a 129S6/SvEvTac (129) background (129-apoE) develop plaques earlier in the aortic arch and later in the aortic root than B6-apoE. Furthermore, *Apoe*-null mice on DBA/2J (DBA) background (DBA-apoE) are highly susceptible to atherosclerosis and develop extensive plaques early at both locations [4]. Our previous analyses of F2 mice derived from an intercross between B6-apoE and 129-apoE mice and an intercross between DBA-apoE and 129-apoE mice demonstrate that most loci that determine susceptibility to atherosclerosis in aortic arch and in aortic roots in these populations are distinct [4, 5, 6]. While QTL identitification in these two crosses provides a list of candidate genes contributing to formation of atherosclerotic lesions, the well known presence of epistatic effects dependent on genetic background in complex phenotypes such as atherosclerosis can not be ignored.

With the aim to ascertain and narrow the candidate intervals of the QTLs influencing atherosclerosis susceptibility in a vascular location dependent manner, we generated, phenotyped and genotyped a F2 population between B6-apoE and DBA-apoE that closes the loop of genetic crosses among three commonly used strains. Comparison of the QTLs mapped in these three crosses confirmed the presence of QTLs that influence atherosclerosis in the aortic arch on chromosome (Chr)2 and on Chr10, and the presence of multiple suggestive QTLs affecting atherosclerosis in the aortic root including QTL on Chr7. Our analyses also revealed that the effects of multiple QTLs appears to depend on the other loci.

## Materials and methods

### Mice

*Apoe*-null mice on DBA and B6 backgrounds were obtained from the Jackson Laboratory (Bar Harbor, ME, USA). *Apoe*-null mice were originally generated in our Laboratory using 129P2/Ola embryonic stem cells [7] and were backcrossed to B6 and subsequently to DBA backgrounds. Male DBA-apoE mice were mated to female B6-apoE mice to generate F1 progeny. F1 hybrids were intercrossed to generate the F2 population. F2 mice (136 females and 96 males) aged 4.5 months were used for analyses in this study. At this age, *Apoe*-null mice on normal chow develop early stage atheromas ranging from monolayers of foam cells to raised plaques with fibrous cap and cholesterol clefts. Plaque sizes rapidly increase and are appropriate for comparison.

Mice were fed regular chow (Prolab IsoProRMH 3000; Agway Inc.) and maintained and handled in accordance with the National Institutes of Health Guide for the Care and the Use of Laboratory Animals under protocols approved by the Institutional Animal Care and Use Committee of the University of North Carolina.

### Quantification of atherosclerotic lesion

Sizes of atherosclerotic plaques in the aortic root and the aortic arch were measured as described previously [5, 8]. Briefly, mice were euthanized with a lethal dose of 2,2,2-tribromoethanol followed by cervical dislocation, and perfused with 4% paraformaldehyde through the left ventricle of the heart. The plaque areas in the defined locations of the aortic arch (S1 Fig) were measured using images captured with Image J software [5]. The plaques at the innercurve of the aortic arch were used to represent “arch plaque size”.

### Plasma lipid analysis

Mice were fasted for 4 h in the morning. Plasma levels of total cholesterol were measured using a commercial kit “Cholesterol E” (Wako Chemicals USA, Richmond, VA). HDL cholesterol levels were measured after removing with magnesium/dextran sulfate apoB containing lipoproteins [9]. Measurements of plasma triglycerides were made using a kit from Stanbio Laboratory (Boerne, TX).

### SNP genotyping

Genomic DNA was isolated from livers of F2 mice using DNeasy Tissue kit (Qiagen, Hilden, German). Genome-wide SNP genotyping was performed with a Mouse Universal Genotyping Array (MUGA) [10]. Samples were processed at the Systems Genetics Core at the University of North Carolina, Chapel Hill and array hybridization was performed by Neogen/Geneseek, Inc (Lincoln, NE).

### QTL analysis

QTL analysis was carried out using R/qtl software. Lesion sizes (in micrometers squared) at the inner curvature of aortic arch and at the aortic root were subject to square root transformation to achieve an approximately normal data distribution. Genotypes from MUGA were prepared for QTL mapping as follows. Markers not informative between B6 and DBA, or with more than 20% missing data among the 232 F2 mice, were removed. Markers in the proximal 20 cM of Chr7 (near the *Apoe* locus) were also removed. Among the remaining markers, a grid of 317 markers spaced at approximately 5 cM intervals across the genome was retained for genetic mapping. Single-locus QTL scans were performed under a model with both additive and dominance effects using the “scanone” function in R/qtl. To account for sex differences, we performed genome scans allowing for either an additive sex effect or (sex—genotype) interaction.

The significance thresholds for LOD scores were determined by 1000 permutations. QTL were considered significant if LOD scores exceeded 95% (p<0.05) of the permutation distribution. They were considered suggestive if the scores exceed 37% (p<0.63) distribution as recommended by the Complex Trait Consortium [11]. Credible interval (CI) was determined from the Bayesian 95% credible interval. For each QTL, mode of inheritance was determined according to allelic effect at the nearest marker of a QTL by performing Haley-Knott regression using the additive and dominant/recessive models as described previously [12, 13]. Percentage of variance was calculated using “fitqtl” function. Potential interactions between two chromosomal loci were examined by “scantwo” function. Pairs with interaction LOD scores >3 represent putative epistatic interactions that were further evaluated by fitting a multiple-QTL model with additive and epistatic terms using “fitqtl”, and then performing model selection by backward elimination with “stepwiseqtl”. Penalties (3.52-additive penalty, 4.28-heavy penalty, 2.69-light interaction penalty) were applied in the “stepwiseqtl” analysis according to recommendations based on simulation from Broman and Sen [14]. QTL data were deposited to QTL Archive at The Jackson Laboratory (https://phenome.jax.org/projects/Maeda1). The physical (Mb) positions (GRCm38) were calculated using Mouse Map Converter tool of the Jackson Laboratory. Haplotype analyses were performed as described [4, 6]. SNPs and nucleotide sequence comparisons of mouse strains were obtained from publicly available resources (http://www.sanger.ac.uk/resources/mouse/genomes/). Estimations of statistical power to determine QTLs and effect size were done using QTL Design Program (http://www.biostat.ucsf.edu/sen/software.html/) [15].

The current cross between B6-apoE and DBA-apoE closes the loop of round-robin crosses, in which each mouse parental strain was crossed with two other strains (129-apoE x B6-apoE, 129-apoE x DBA-apoE, B6-apoE x DBA-apoE). Therefore, we compared the findings of this QTL analysis with results from two previous F2 crosses of 129-apoE x DBA-apoE and B6-apoE x 129-apoE.

### Haplotype analysis

The haplotype patterns of genomes in the QTL interval were analyzed using the Mouse Phylogeny Viewer (http://msub.csbio.unc.edu/) [16]. The likelihood that an amino acid change is detrimental to a protein was examined by Sorting Intolerant From Tolerant (SIFT) program (http://sift.jcvi.org/) [17] and PROVEAN (http://provean.jcvi.org/) [18]. Gene expression in the aorta of wild type B6, DBA and 129 were previously published (GEO accession number -GSE53006) [4]. RNA from three aortic arch samples per strain, each pooled from 3–5 mice, were hybridized on a Mouse Gene 2.1 ST 24- Array plate (Affymetrix). For eQTL analysis, aortic gene expression in the Hybrid Mouse Diversity Panel (HMDP) was used (GSE38120, Bennett BJ et al, 2010) [19]. SNPs were picked from the eQTL data of the HMDP according to the following criteria: (1) location within and near the QTL interval; (2) being associated with genes at p < 1.0 ×10^−6^.

### Statistical analysis

Trait data were analyzed using JMP software version 8.0 (SAS Institute, Cary, NC). Comparisons of multiple groups in trait values and gene expression levels in the aorta were done by one-way analysis of variance (ANOVA), followed by Tukey-Kramer’s HSD-test. Transformation of values for each phenotype in F2 mice was assessed for normality by Shapiro–Wilk test and Kolmogorov.

## Results

### The correlation between lesion size and plasma lipid levels is low in B6-apoE x DBA-apoE F2 mice

Size of atherosclerotic lesions at age 4.5 months at the aortic root and the aortic arch, plasma lipid concentrations in parental and F2 mice are summarized in Table 1. Parental DBA-apoE mice develop significantly larger atherosclerotic plaques at both the aortic root and the innercurve of aortic arch than B6-apoE mice. Briefly, the mean root lesion in DBA-apoE mice was 17 times larger in males and four times larger in females compared with gender and age-matched B6-apoE mice. Similarly, arch lesion size in DBA-apoE mice was 26 times greater in males and 14 times greater in females compared with B6-apoE mice. Notably, while DBA-apoE mice did not show significant sex differences in plaque size at either location, B6-apoE females had significantly larger plaques than B6-apoE males at the aortic root, but not at the aortic arch, that are consistent with our previous study [5].

**Table 1:** Atherosclerotic lesion sizes and plasma lipid levels in the parental mice and F2 from intercross between DBA-apoE and B6-apoE mice.

	Sex	B6-apoE (n)	DBA-apoE (n)	F2(n)
Root lesion (10^4^ μm^2^)	Male	2.6±0.4 (10)	43.7±2.4 (13) ^c^	19.0±1.3 (91)
	Female	12.8±2.3 (8) ^d^	51.9±3.1 (13) ^c^	33.6±1.4 (129) ^e^
Arch lesion (10^4^ μm^2^)	Male	5.2±1.0 (10)	133.0 ± 6.8 (16) ^c^	76.6±3.8 (96)
	Female	9.1±2.8 (10)	129.3 ± 6.1 (16) ^c^	71.2±2.7 (134)
Cholesterol (mg/dl)	Male	200±12(10)	671±29 (18) ^c^	705±16 (96)
	Female	189±9 (10)	665±48 (18) ^c^	573±11 (136) ^e^
HDL-Cholesterol (mg/dl)	Male	45.9±6.7 (10)	61.4±6.0 (18)	48.8±1.9 (96)
	Female	39.2±2.1(10)	57.8±3.3 (18) ^b^	38.6±1.7 (134) ^e^
Triglycerides (mg/dl)	Male	82±12(10)	186±22 (18)^a^	179±10 (96)
	Female	63±6 (8)	166±19 (18) ^c^	76±3 (135) ^e^

Data are shown as the mean ± SE. Data of the DBA-apoE mice are from our previous papers [4, 6]. The number of mice is shown in parentheses. Statistical comparisons between groups were performed using Student's t test.

^a^ p<0.001,

^b^ p<0.0001,

^c^ p<0.00001 vs B6-apoE within each sex;

^d^ p<0.001 B6-apoE female vs B6-apoE male;

^e^ p<0.0001 F2 female vs F2 male.

At the aortic root, both male and female F2 mice had intermediate lesion size between the parental strains, and female F2 mice showed about twice larger plaque size than male F2 mice. Plaques at the aortic arch of of F2 mice were also intermediate between the parental strains, but no sex difference was observed. On a per animal basis, there was no correlation between the size of the aortic lesions at the root and the arch (r = 0.12, p = 0.07).

Hyperlipidemia is a well-known cause of the development of atherosclerosis. DBA-apoE mice had significantly higher plasma total cholesterol (p<0.00001), HDL-cholesterol (p<0.0001 in female) and triglycerides (p<0.001 in male, p<0.00001 in female) concentrations than B6-apoE mice. Average plasma total cholesterol and triglycerides in F2 mice were similar to those in the parental DBA-apoE mice (Table 1). When we examined the relationship between plasma lipid concentrations and plaque size at two vascular locations, only the total cholesterol level in males significantly correlated with root lesion size (r = 0.32, p = 0.002) (S2 and S3 Figs). Thus, plasma lipid level does not appear to be a strong determinant of atherosclerotic plaque size in this F2 population of *Apoe*-null mice.

### Analysis of B6-apoE x DBA-apoE F2 population reveals a significant QTL on Chr2 and a suggestive QTL on Chr10 which influence aortic arch lesion size

**Fig 1:**
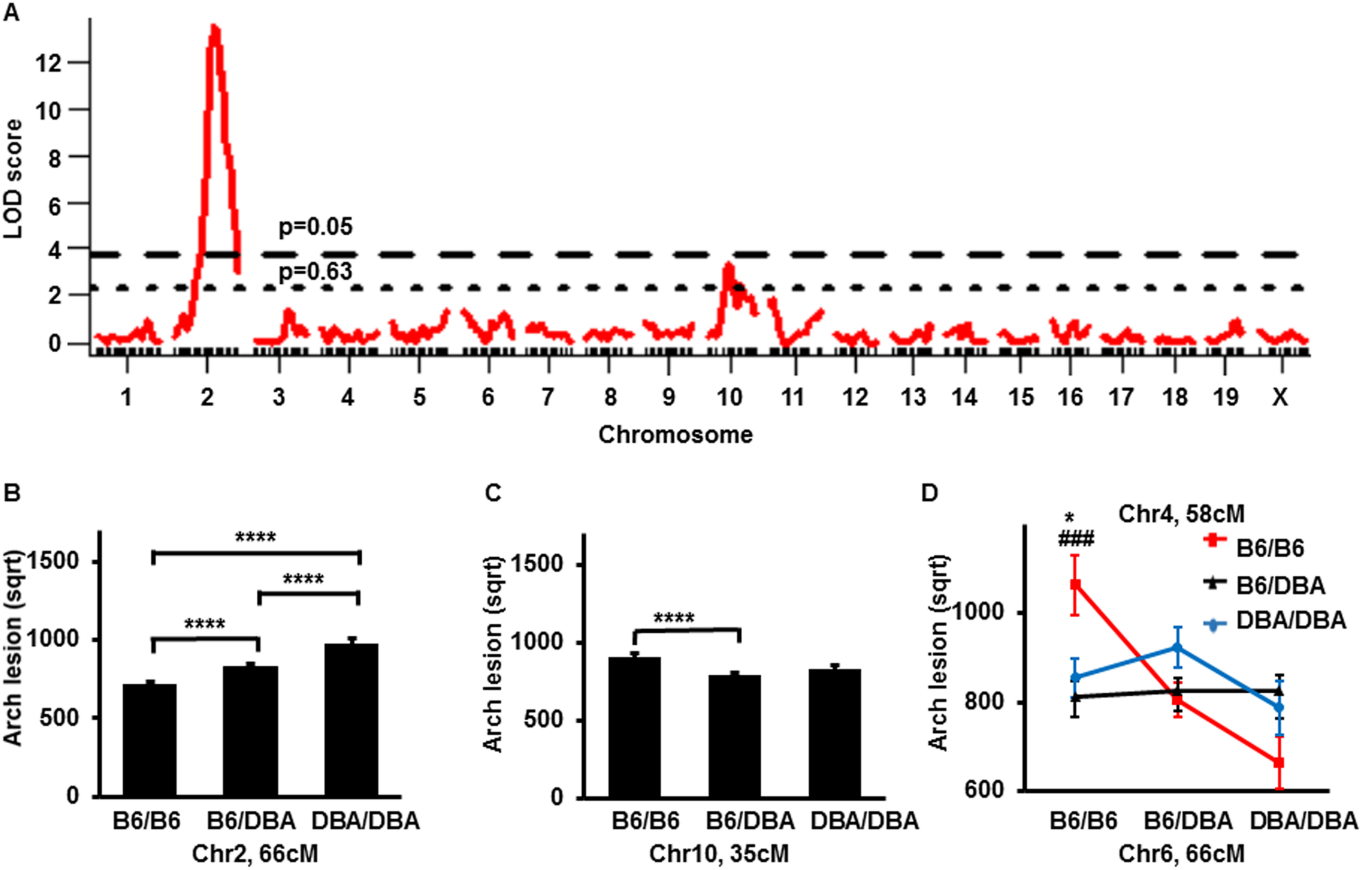
LOD curves and allelic distribution of QTLs for arch plaque lesion. (A) LOD curve for arch lesion size with sex as additive covariate in F2 mice from a cross B6-apoE x DBA-apoE. The horizontal dashed line represents a threshold for a suggestive (p = 0.63) QTL and a dotted line represents a threshold for a significant QTL (p = 0.05). The significance thresholds for LOD scores were determined by 1000 permutations using R/qtl software. (B) Allelic distribution of the QTL on Chr2 for plaque lesion size at the arch at the nearest marker to the peak in F2 mice from the cross B6-apoE x DBA-apoE. Data represent mean ± SE. Lesion size comparison was performed by one-way analysis of variance (ANOVA) (p<0.0001) followed by Tukey-Kramer’s HSD-test. **** p<0.0001 (C) Allelic distribution of QTL on Chr10 for plaque lesion size at the arch at the nearest marker to the peak in F2 mice from the cross B6-apoE x DBA-apoE. Data represent mean ± SE. Lesion size comparison was performed by one-way analysis of variance (ANOVA) (p<0.001) followed by Tukey-Kramer’s HSD-test. p = 0.06 between B6/B6 and DBA/DBA genotypes, **** p<0.0001 between B6/B6 and B6/DBA genotypes (D) Allelic distribution of the main effect QTL for plaque lesion size at the arch in Chr 4 and Chr 6 in both sexes in F2 mice from the cross B6-apoE x DBA-apoE. Data represent mean ±SE. Only mice homozygous for B6 allele at both Chr4 and Chr6 have increased plaque size by one-way ANOVA (p = 0.001), followed by Tukey-Kramer’s HSD-test: * p<0.05- B6/B6 genotype vs DBA/DBA genotype, ### p<0.001- B6/B6 genotype vs B6/DBA genotype.

A genome-wide QTL scan for atherosclerotic lesions at the aortic arch was performed in 232 F2 mice, using sex as an additive (Fig 1A, S4A and S4B Fig, Table 2, S1 Table) or interactive covariates (S4A and S4C Fig). A scan in sex-additive model identified a highly significant QTL for aortic arch lesion on Chr2 (peak 66 cM, CI = 60–71 cM, LOD = 13.6) accounting for 21.1% of the variance. At the QTL peak, the DBA allele was associated with bigger arch lesion size than the B6 allele, and had an additive effect (Fig 1B). The QTL on Chr2 influenced plaque sizes at all locations of the aortic arch as shown in the S1 Table. The sex-additive scan also detected a suggestive QTL on Chr10 (peak 35 cM, CI = 28–69 cM, LOD = 3.4), which accounts for 4.0% of the variance. The B6 allele at the Chr10 QTL was associated with an increased plaque size at the aortic arch and was recessive with respect to the DBA allele (Fig 1C). The QTL at Chr10 was also suggestive for plaque size at the branching point of the innominate artery but not other branches (S1 Table). These features are consistent with the assignment of QTLs on Chr2 and Chr10 as *Aath4* and *Aath5* respectively, that we previously identified in the F2 population from DBA-apoE x 129-apoE [6] as discussed below.

**Table 2:** QTLs for atherosclerosis at the aortic arch in F2 mice from intercross between DBA-apoE and B6-apoE.

	Chr	Peak (cM)	CI (cM)	Peak (Mb)	CI (Mb)	LOD	Significance	High allele	Mode of inheritance	%
Arch										
Female+male	2	66	60–71	135	119–143	13.6	Significant	DBA	Additive	21.1
	10	35	28–69	66	55–121	3.4	Suggestive	B6	Overecessive	4.0
Female	2	61	58–70	105	96–128	8.5	Significant	DBA	Recessive	25.1
Male	2	67	59–80	155	135–170	5.6	Significant	DBA	Additive	19.2
	10	35	29–72	79	61–124	3.1	Suggestive	B6	Recessive	9.6

CI, 95% credible interval; LOD score for sex-combined scan shown in Table 2 was determined in a single locus scan using sex as additive; the significance thresholds for LOD scores were determined by 1000 permutations using R/qtl software; for each QTL, a model of inheritance was determined according to allelic effect at the nearest marker of a QTL by performing Haley-Knott regression using the additive and dominant/recessive models; % Variance indicates the percentage of the total F2 phenotypic variance.

We next assessed the potential interactions among different chromosomal regions by performing a two-dimensional genome scan. One of the epistatic interactions identified in this scan was an interaction between Chr4 at 58 cM and Chr6 at 66 cM (Fig 1D). The phenotype of mice that are homozygous for the B6 allele on Chr4 depends on their genotype on Chr6: only mice homozygous for B6 at both Chr4 and Chr6 have increased plaque size, while mice with opposite homozygous combinations have decreased plaque size. However, this interaction did not meet the genome-wide threshold for significance in F2 cross of this size, after accounting for multiple testing.

### Comparison of QTLs for arch atherosclerosis from three F2 intercrosses of *Apoe*-null mice on B6, DBA and 129 backgrounds

**Fig 2:**
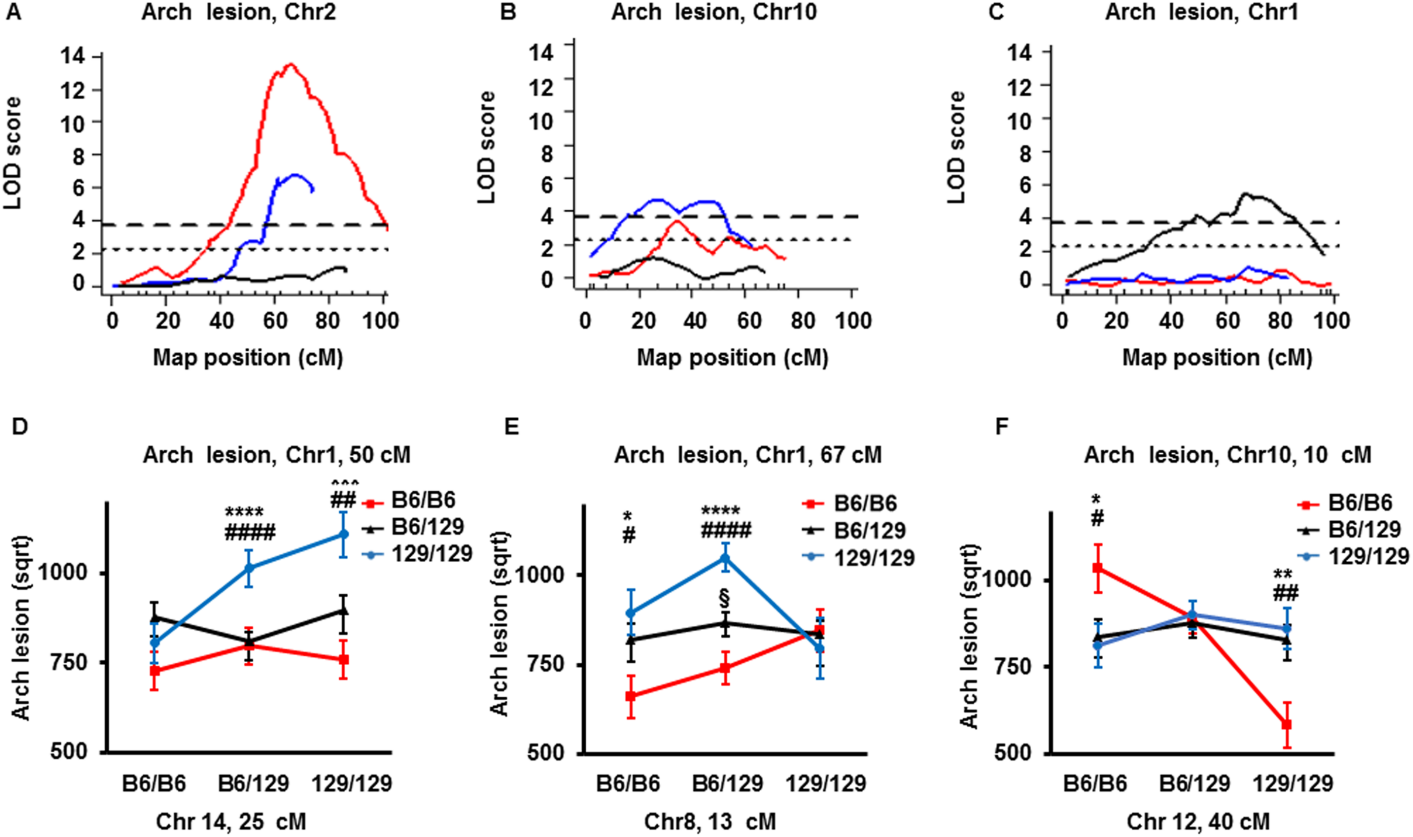
LOD curves and allelic distribution of QTLs for plaque lesion at arch. (A-C) LOD curves of QTL for plaque lesion size at arch in sex-combined scan with sex as additive covariate of a cross between B6-apoE and DBA-apoE (red lines), a cross between B6-apoE and 129-apoE mice (black lines) and a cross between DBA-apoE and 129-apoE mice (blue lines) on chromosome Chr2 (A), Chr10 (B) and Chr1 (C). The horizontal dashed and dotted lines represent thresholds for suggestive QTL (p = 0.63) and significant QTL (p = 0.05) determined in single locus scan of the cross between B6-apoE x DBA-apoE mice. The significance thresholds for LOD scores were determined by 1000 permutations using R/qtl software. (D) Allelic distribution of the QTLs for plaque lesion sizes at arch at the nearest marker to the peaks in Chr1 and Chr14 in both sexes in the cross between B6-apoE and 129-apoE mice. Data represent mean ± SE. Comparison of lesion sizes were done by one-way analysis of variance (ANOVA), followed by Tukey-Kramer’s HSD-test. Only mice homozygous for 129 allele at both Chr1 and Chr14 have significantly larger plaques compared to those with other combinations of the loci by one-way ANOVA (p = 0.001), followed by Tukey-Kramer’s HSD-test: *** p<0.001–129/129 genotype vs B6/B6 genotype, ^##^ p<0.01–129/129 vs 129/B6 genotype genotype. **** p<0.0001–129/129 genotype vs B6/B6 genotype, ^####^ p<0.0001–129/129 genotype vs B6/129 genotype; (E) Allelic distribution of the QTLs for plaque lesion sizes at arch at the nearest marker to the peaks in Chr1 and Chr8 in both sexes in the cross between B6-apoE and 129-apoE mice. If Chr8:13 cM is homozygous for the 129 allele, genotype effects were not present, but that if at least one allele of Chr8;13cM derived from B6, a strong effect of genotypes is revealed. Data represent mean ± SE. Comparison of lesion sizes were done by one-way analysis of variance (ANOVA), followed by Tukey-Kramer’s HSD-test. * p<0.05–129/129 genotype vs B6/B6 genotype; ^#^ p<0.05–129/129 genotype vs B6/129 genotype, **** p<0.0001–129/129 genotype vs B6/B6 genotype; ^####^ p<0.0001–129/129 genotype vs B6/129 genotype; § p<0.05- B6/B6 genotype vs B6/129 genotype. (F) Allelic distribution of the QTLs for plaque lesion sizes at arch at the nearest marker to the peaks Chr10 and Chr12 in both sexes in the cross between B6-apoE and 129-apoE mice. Data represent mean ± SE. 129 allele of the Chr12 has significant protective effect only when Chr10 is homozygous for B6 allele Comparison of lesion sizes were done by one-way analysis of variance (ANOVA), followed by Tukey-Kramer’s HSD-test. * p<0.05- B6/B6 genotype vs 129/129 genotype; ^#^ p<0.05- B6/B6 genotype vs B6/129 genotype; **p<0.01- B6/B6 genotype vs 129/129 genotype; ^##^ p<0.01- B6/B6 genotype vs B6/129 genotype.

We next compared QTLs for arch atherosclerosis that were significant at least in one of the F2 populations (Table 3). Since a cross between B6-apoE x DBA-apoE mice, a cross between B6-apoE and 129-apoE mice and a cross between DBA-apoE and 129-apoE mice were made from mice on three inbred backgrounds in a round-robin fashion, given large-effect QTLs are expected in a simple case to be detected at least in two crosses, because every genetic variant was brought at least twice. This pattern was observed in two of the three significant QTLs for arch plaque sizes. Thus the significant QTL at Chr2:66 cM (CI = 60–71 cM) in the current B6-apoE x DBA-apoE (red line) overlaps with *Aath4*, a significant QTL at Chr2:68 cM (CI = 61–73 cM) identified in the DBA-apoE x 129-apoE cross (blue line) (Fig 2A). No peak was present in the same region of Chr2 in the F2 population from the B6-apoE x 129-apoE cross (black line). This relationship confirms that the DBA allele of *Aath4* contributes to atherosclerosis susceptibility, and that the genomic features that are unique in DBA, but are shared in 129 and B6, are responsible for this susceptibility. We previously reported genes having this feature as *Aath4* candidates, including *Mertk* that is important for the clearance of apoptotic cells and *Siglec1* that is involved in mediating cell-cell interactions [6].

**Table 3:** QTLs for atherosclerotic lesions at arch in three F2 intercrosses of Apoe-null mice on B6, DBA and 129 backgrounds.

QTL	Chr	Peak (CI), cM	B6-apoE x DBA-apoE	DBA-apoE x 129-apoE	B6-apoE x 129-apoE	Consistency
*Aath1*	1	49 (41–63)	-	-	**129>B6, add**	No
*Aath2*	1	69 (63–79)	-	-	**129>B6, add**	No
*Aath3*	15	46 (38–57)	-	-	**129>B6, add, F**	No
*Aath4*	2	72 (61–73)	**DBA>B6, add**	**DBA>129, add**	-	129=B6≠DBA
*Aath5*	10	26 (17–52)	B6>DBA, ovr	**129>DBA, dom**	-	129=B6≠DBA
	12	34 (26–43)	-	-	B6>129, dom, M	No
	13	17 (6–23)	-	-	129>B6, add, F	No
	19	34 (17–39)		129>DBA, rec, M		No

QTL, quatitative trait locus: Chr, chromosome; CI, 95% credible interval; add, additive; dom, dominant; rec, recessive; ovr, overrecessive: F-female only scan; M- male only scan; significant QTLs are shown in bold, other QTLs are suggestive. > shows allelic relationship. For example, 129>B6 indicates that F2 mice homozygous for 129 allele at the locus develop larger plaque than those with B6 allele.

Similarly, the QTL at Chr10:35 cM (CI = 28–69 cM) of the current B6-apoE x DBA-apoE cross overlaps with *Aath5* at 26 cM (CI = 17–52 cM) which was significant in the DBA-apoE x 129-apoE cross (Fig 2B). The peak was not detected in the B6-apoE x 129-apoE cross, confirming that the genomic features that are common in 129 and B6, but differ from DBA in this QTL region, confer susceptibility to arch atherosclerosis. While CIs of both QTLs are wide and the presence of more than two loci cannot be eliminated completely, atherosclerosis susceptibility loci likely reside within the range of 28–52 cM on this chromosome. Candidate genes of *Aath5*, including a scavenger receptor, *Stab2*, were previously discussed [6].

In contrast, significant QTLs for arch atherosclerosis, *Aath1*, *Aath2* and *Aath3*, found in the B6-apoE x 129-apoE cross, showed no corresponding peaks in either of the other two crosses (Fig 2C, Table 3), suggesting that some other chromosomal loci are interacting with these QTLs. We therefore examined possible interactions in the B6-apoE x 129-apoE cross. Scan-two analysis detected that *Aath1* on Chr1 probably interacts with Chr14. Thus, mice homozygous for the 129 alleles of both Chr1 at 50 cM and Chr14 at 25 cM have significantly larger plaques compared to those with other combinations of the loci (Fig 2D).

Scan-two analysis also showed that *Aath2* at Chr1:67 cM interacts with Chr8 at 13 cM in such a way that when Chr8 is homozygous for the 129 allele, no *Aath1* genotype effects were present, but that if at least one allele of Chr8:13 cM is derived from B6, a strong effect of *Aath2* genotype is revealed (Fig 2E). Since *Aath2* peak is present in the B6-apoE x 129-apoE cross, its candidates must be where 129 sequences differ from that of B6. If the *Aath2* allele of DBA shares sequence with B6, the Chr8:13 cM sequence must be shared between 129 and DBA to explain the absence of the peak in the DBA-apoE x 129-apoE cross. If DBA and 129 share the same allele at *Aath2*, then 129 and DBA are likely to share alleles at Chr8:13 cM to explain the absence of the peak in the B6-apoE x DBA-apoE cross. Thus although our round-robin crosses do not narrow the *Aath2* interval, its potential interaction with Chr8:13 cM could help identify the causative variants underlying *Aath2*. Interestingly, Chr10:10 cM interacts with Chr12:40 cM significantly, and that 129 allele of the Chr12 has significant protective effect only when Chr10 is homozygous for B6 allele (Fig 2F). To further evaluate loci interactions, we performed genome-wide scans allowing for pair-wise interactions. “Stepwiseqtl” search algorithm confirmed significant interaction between Chr12 and Chr10 (pLOD = 4.6).

### The suggestive QTLs for root plaque sizes were detected in the B6-apoE x DBA-apoE F2 population

**Fig 3:**
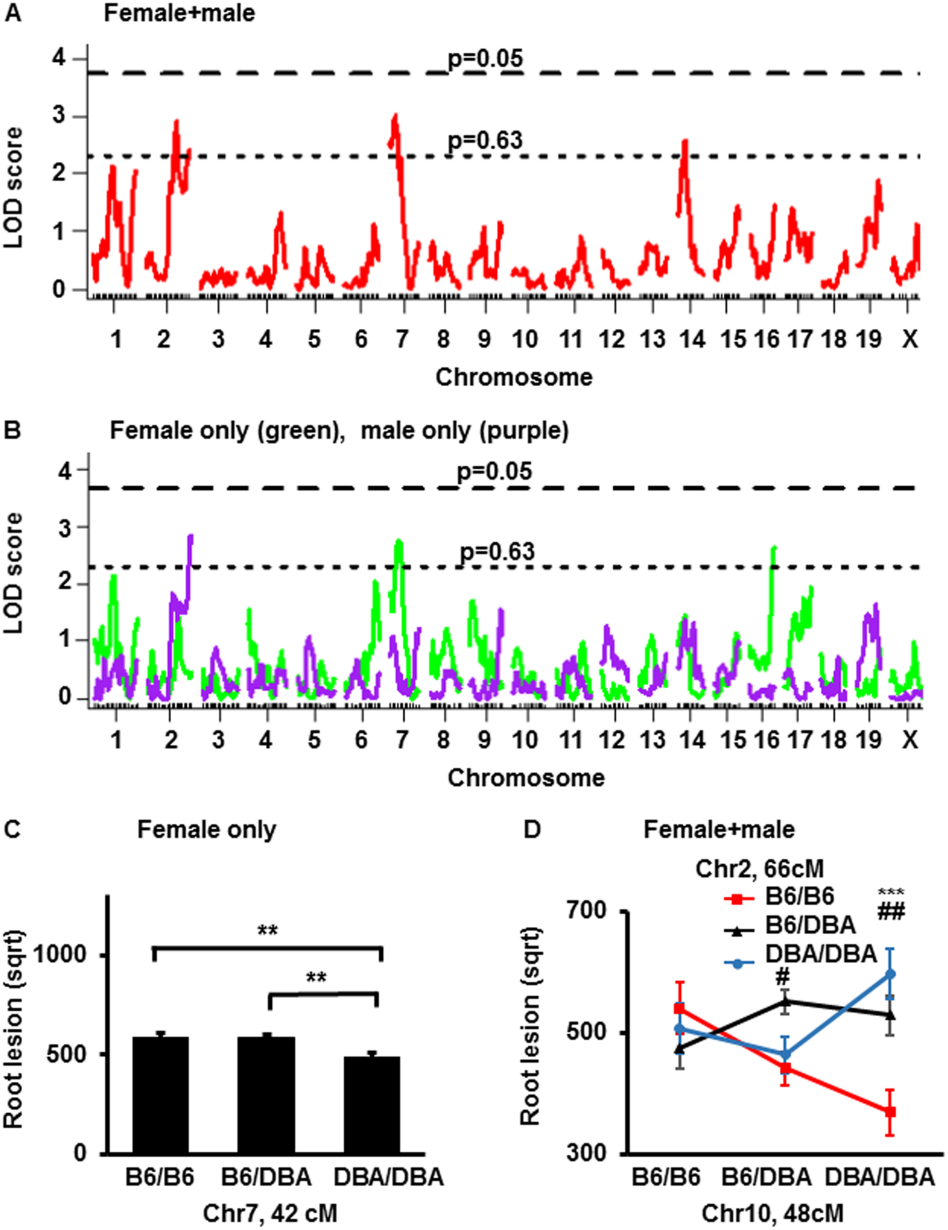
LOD curves and allelic effect of QTL for root plaque size. (A) LOD curve for root lesion size with sex as additive covariate in F2 mice from a cross B6-apoE X DBA-apoE. The horizontal dashed and dotted lines represent thresholds for suggestive (p = 0.63) and significant (p = 0.05) QTLs. The significance thresholds for LOD scores were determined by 1000 permutations using R/qtl software. (B) A LOD curve for root lesion size in female-only (green) and male-only (purple) scans in F2 mice from cross B6-apoE X DBA-apoE. The horizontal dashed and dotted lines represent thresholds for suggestive (p = 0.63) and significant (p = 0.05) QTLs. The significance thresholds for LOD scores were determined by 1000 permutations using R/qtl software. (C) Allelic distribution of the main QTLs for plaque lesion size at root at the nearest marker to the peak at Chr 7 in female F2 mice from a cross B6-apoE X DBA-apoE. Data represent mean ±SE. Comparison of lesion sizes were done by one-way analysis of variance (ANOVA) (p<0.01) followed by Tukey-Kramer’s HSD-test, **p<0.01 (D) Allelic distribution of the main effect QTLs for plaque size (μm^2^) subjected to square root transformation (sqrt) at the root in Chr 4 and Chr 6 in both sexes in F2 mice from cross B6-apoE X DBA-apoE. Data represent mean ±SE. Comparison of lesion sizes were done by one-way analysis of variance (ANOVA), followed by Tukey-Kramer’s HSD-test. *** p = 0.001- B6/B6 genotype vs DBA/DBA genotype; # p<0.05- B6/B6 genotype vs B6/DBA genotype, ## p<0.01- B6/B6 genotype vs B6/DBA genotype.

A single locus scan for QTLs for atherosclerotic lesions at the aortic root using sex as an additive covariate detected suggestive QTLs on Chr2 (peak 74 cM, CI = 58–103 cM, LOD = 2.9,), Chr7 (peak 37 cM, CI = 23–48 cM, LOD = 3.0)) and Chr14 (peak 22 cM, CI = 7–32 cM, LOD = 2.6), contributing 6.8, 5.0 and 4.9% to the variance, respectively (Fig 3A, S5A and S5B Fig, Table 4). Single-QTL genome scan for root plaque size with sex as an interactive covariate determined suggestive QTL on Chr7 (S5A and S5C Fig). Consistent with our earlier observations in the B6-apoE x 129-apoE and DBA-apoE x 129-apoE crosses, the QTLs for the aortic atherosclerosis and root atherosclerosis in the F2 from B6-apoE x DBA-apoE were largely independent [4,5,6]. Since the root plaque sizes were significantly larger in B6-apoE females than in B6-apoE males, we also performed a single locus scan in males and females separately (Fig 3B, Table 4). Male only analysis revealed a suggestive QTL on Chr2 (peak 102 cM, CI = 57–103 cM, LOD = 2.9), accounting for 13.4% of the variance in root plaque size. The QTL scan among females only, on the other hand, revealed suggestive QTLs on Chr7 (peak 42 cM, CI = 23–52 cM, LOD = 2.8) and on Chr16 (peak 58 cM, CI = 3–58 cM, LOD = 2.7) which accounts for 8.0 and 7.6% of variance in root plaque size, respectively. The B6-allele on Chr7 is associated with a larger root plaque size compared with DBA allele, and has an additive effect (Fig 3C).

**Table 4:** QTLs for atherosclerosis at the aortic root in F2 mice from intercross between DBA2-apoE and B6-apoE.

	Chr	Peak (cM)	CI (cM)	Peak (Mb)	CI (Mb)	LOD	Significance	High allele	Mode of inheritance	%
Root										
Female+male	2	74	58–103	149	114–181	2.9	Suggestive	DBA	Dominant	6.8
	7	37	23–48	68	37–84	3.0	Suggestive	B6	Additive	5.0
	14	22	7–32	40	16–61	2.6	Suggestive	B6	Recessive	4.9
Female	7	42	23–52	71	37–89	2.8	Suggestive	B6	Dominant	8.0
	16	58	3–58	94	56–94	2.7	Suggestive	DBA	Additive	7.6
Male	2	102	57–103	189	131–193	2.9	Suggestive	DBA	Additive	13.4

CI, 95% credible interval; LOD, logarithm of odds; LOD score for sex-combined scan shown in Table 4 was determined in single locus scan using sex as additive; the significance thresholds for LOD scores were determined by 1000 permutations using R/qtl software; for each QTL, model of inheritance was determined according to allelic effect at the nearest marker of a QTL by performing Haley-Knott regression using the additive and dominant/recessive models; % Variance indicates the percentage of the total F2 phenotypic variance.

A search for two-locus interactions identified a putative interaction between Chr2 at 66 cM and chr10 at 48 cM contributing to 7.3% of the phenotypic variance (LOD-interaction = 3.8). This relationship suggests the DBA allele at Chr10 has divergent effects depending on genotype at Chr2 (Fig 3D). However, as for plaque size at the aortic arch, the Chr2-Chr10 interaction did not reach the threshold for genome-wide significance after accounting for multiple testing.

### Comparisons of three crosses verify suggestive QTLs on Chr7 for aortic root plaque size

**Fig 4:**
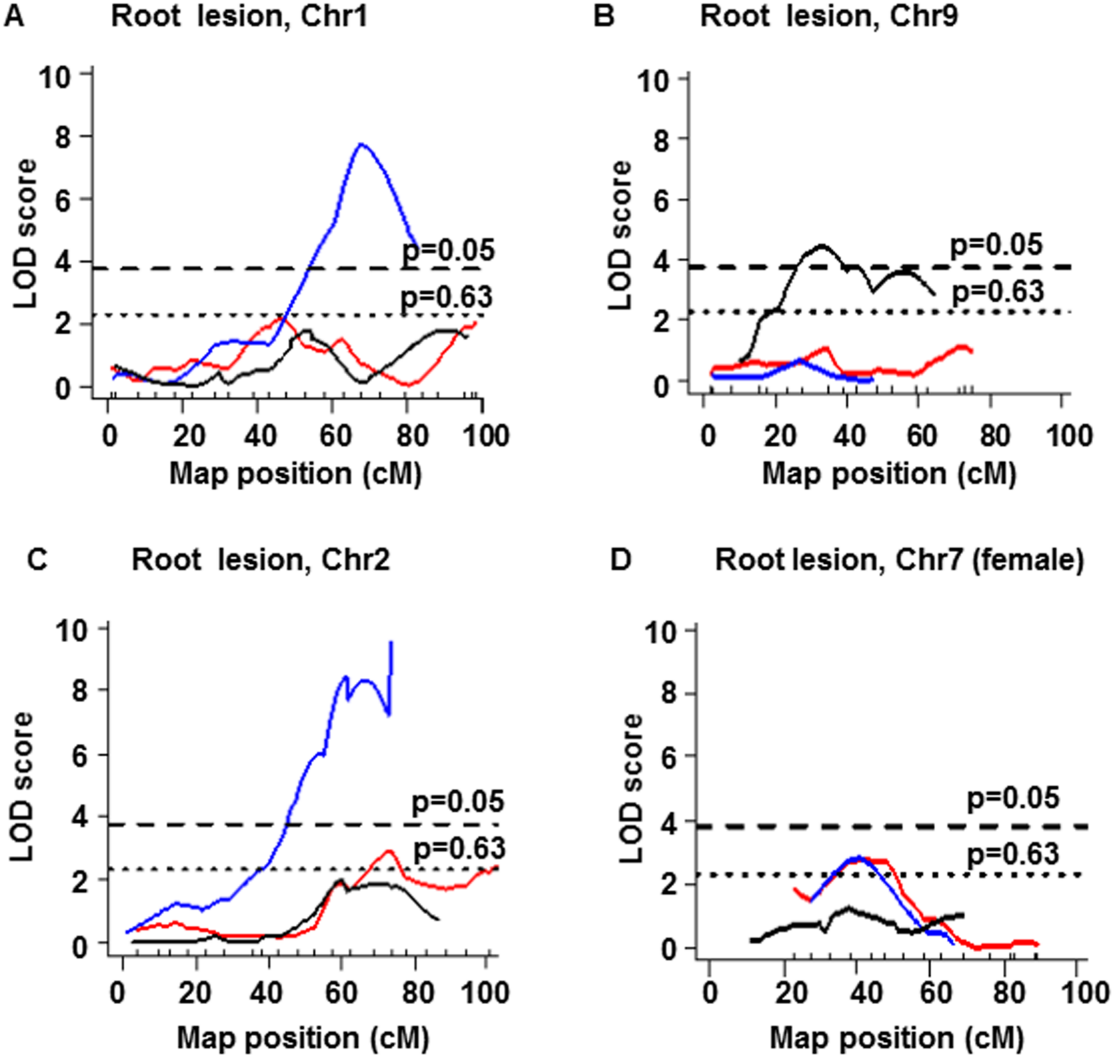
LOD curves of QTLs for plaque lesion size at root. (A-D) LOD curves of QTL for plaque lesion size at root in sex-combined (A,B, C) scans on Chr1 (A), Chr9 (B), Chr2 (C) and female-only (D) scan on Chr7 (D) cross between B6-apoE and DBA-apoE (red lines), cross between B6-apoE and 129-apoE mice (black lines) and cross between DBA-apoE and 129-apoE mice (blue lines). The horizontal dashed and dotted lines represent thresholds for suggestive QTL (p = 0.63) and significant QTL (p = 0.05) determined in single locus scan of crosses between B6-apoE x DBA-apoE mice. The significance thresholds for LOD scores were determined by 1000 permutations using R/qtl software.

Surprisingly, strong QTLs for aortic root plaques were identified only in one of the three round-robin crosses. Thus, highly significant QTL peak, *Ath44*, on Chr1 at 68 cM was present only in the DBA-apoE x 129-apoE F2 population (Fig 4A, Table 5). Similarly, the root plaque QTL on Chr9 at 33 cM was highly significant in the B6-apoE x 129-apoE F2, but was not detected either in the B6-apoE x DBA-apoE or in the DBA-apoE x 129-apoE F2 populations (Fig 4B). No significant interactions with these loci or other chromosomal locations were evident. *Ath45* on Chr2 also appears complex, since it was highly significant in the DBA-apoE x 129-apoE F2 population, but suggestive peaks were present in the other two crosses (Fig 4C, Table 5). Multiple genetic variants may be affecting atherosclerosis in this chromosomal location.

**Table 5:** QTLs for atherosclerotic lesions at root in three F2 intercrosses of Apoe-null mice on B6, DBA and 129 backgrounds.

QTL	Chr	Peak (CI), cM	B6-apoE x DBA-apoE	DBA-apoE x 129-apoE	B6-apoE x 129-apoE	Consistency	Overlap
*Ath44*	1	68 (64–74)	-	**DBA>129, rec**	-	No	*Ath1*
*Ath45*	2	83 (78–85)	DBA>B6, add	**DBA>129, add**	B6>129, dom, M	?	*Athla1*
*Ath29*	9	33 (25–39)	-	-	**B6>129, add, M**	No	*Ath29*
*Ath31*	7	41 (31–50)	B6>DBA, dom, F	129>DBA, dom, F	-	129=B6≠DBA	*Ath3*, *Aorls2* *Ath31*

QTL, quatitative trait locus: Chr, chromosome; CI, 95% credible interval., add, additive; dom, dominant, rec, recessive; F-female only scan, M- male only scan; significant QTLs are shown in bold, other QTLs are suggestive. > shows allelic relationship. For example, DBA>129 indicates that F2 mice homozygous for DBA allele at the locus develop larger plaque than those with 129 allele.

Among the QTLs for root plaques, however, a QTL on Chr7 was suggestive in B6-apoE x DBA-apoE F2 population in both sex-combined (peak at 37 cM) and female-only (peak at 42 cM) scans. This QTL overlaps with a suggestive QTL on Chr7 found in DBA-apoE x 129-apoE F2 mice (Table 5). The peak was absent at the corresponding location in B6-apoE x 129-apoE F2 mice (Fig 4D). This suggests that the DBA allele of the QTL within 31–48 cM of Chr7 is athero-protective over the allele shared by B6 and 129. Further analysis showed that there are 51 genes at which B6 mice share a haplotype with 129, but differ from DBA mice. Twenty five genes contain amino acid substitutions, of which 10 are predicted as potentially damaging by SIFT and/or PROVEAN prediction programs (S3 Table). In addition to qualitative changes in the protein structure, genetic variants could also affect quantitatively the amount of gene products. Analysis of our previous microarray data of gene expression in the aortas of wild type B6, 129 and DBA mice (GEO accession number- GSE53006) [6] found several genes whose expression in DBA aortas are either higher or lower than in 129 and B6 (S4 Table). Chr7: 62.5–65 Mb is also remarkable that the SNPs in this region are associated significantly with the expression of multiple genes in trans. Together, we narrowed down the list of candidate genes on Chr7 that may contribute to formation of atherosclerotic plaques, which include *Atp10a*, *Pcsk6* and *Mctp2*. SNPs in or near these genes are associated with cardiovascular diseases in humans [20, 21, 22].

## Discussion

To determine the genetic factors that affect susceptibility to atherosclerosis in a vascular location-dependent manner, we performed genome wide scans for QTLs in F2 progeny from an intercross of *Apoe*-null mice on B6 and DBA backgrounds. Our study revealed a significant QTL on Chr2 (peak 66 cM) and a suggestive QTL on Chr10 (peak 35 cM) that influence aortic arch lesion. These QTLs respectively overlap with *Aath4* and *Aath5* that were previously found in the F2 population from a cross of *Apoe*-null mice on DBA and 129 backgrounds. For the plaques at the aortic roots, we identified only suggestive QTLs. Among them a QTL on Chr7 at 37 cM also overlaps with a suggestive QTL found in the cross between the *Apoe*-null mice on DBA and 129 backgrounds. Since none of these QTLs were found in the cross between B6-apoE and 129-apoE, our data confirms that DBA specific sequences in these QTLs influence atherosclerosis susceptibility positively or negatively over the 129 and B6 sequences.

The current cross closes the circle of F2 populations generated using round-robin design from *Apoe*-null mice on three genetic backgrounds, 129, B6 and DBA (129-apoE x B6-apoE, 129-apoE x DBA-apoE, B6-apoE x DBA-apoE). Genetic analysis of round-robin crosses helps to validate some QTLs, identify epistaticaly interacting loci and narrow the list of candidate genes. Using round-robin design in our three crosses, we expected QTLs with large additive effects and strong penetrance to be detectable in two out of the three crosses, because every genetic variant was brought at least two times. Indeed, confirmation of the three QTLs above assures that the inheritance of these loci is likely to be simple, and provides an important step towards our goal of identifying genetic variants that influence atherosclerosis susceptibility differently at different aortic locations. In our earlier study, we have predicted that the DBA specific variants of *Aath4* on Chr2 and *Aath5* on Chr10 are likely responsible for the susceptibility and protection, respectively, for the aortic arch plaques [6]. Our current study fully supports these predictions.

Surprisingly, however, many of the QTLs were significant only in one of the crosses and lacked even suggestive peaks in the other two crosses. Observation of QTL in one cross only can be explained by absence of statistical power, epistasis or others factors [23]. Certainly, statistical power to determine QTLs is affected by a sample size (number of animals in F2 population) and effect size. Using QTL Design Program, we estimated that for effect size 20X10^4^ μm^2^ statistical power to determine QTLs for atherosclerosis at aortic arch in our cross B6-apoE x DBA-apoE (n = 232) is about 0.99 if genetic variance is modest. However, minimum detectable effect size (power = 0.8) for atherosclerotic lesion at arch in this cross is about 15X10^4^ μm^2^ if mode inheritance is additive. Therefore, relatively small sample size of F2 population (232 mice) in our study may result in our failure to determine QTLs with small effect size (>15X10^4^ μm^2^ for arch lesion) and overestimation of effect size of detected QTLs (“Beavis effect”) [24, 25]. Another explanation for the background effect is the presence of gene-gene interaction (epistasis). *Aath1* and *Aath2* on Chr1 for aortic arch plaques may represent such an example, since we have detected possible interactions, including, *Aath1* with Chr14, and *Aath2* with Chr8. This could explain our failure to detect the particular QTLs in other crosses than in B6-apoE x 129-apoE. These interactions were, however, not significant in “stepwise” analysis. Furthermore, there were no suggestive interactions revealed involving *Aath3* on Chr15, a female specific aortic arch lesion QTL [5]. Similarly, no interactions were found involving root plaque significant QTLs on Chr9 in cross between B6-apoE and 129-apoE or on Chr1 in B6-apoE x DBA-apoE cross [4, 5]. Interactions between more than two loci are also possible but were not examined since the numbers of samples used in these studies severely limits the power of scans for multiple epistasis.

The presence of multiple loci affecting atherosclerosis development could result in non-detectable QTL peak at a certain locus in some crosses when their combined effects become negligible. This is likely, since each QTL has a broad credible interval that can contain genomic regions with mixed haplotype sharing patterns. It could also explain the *Ath45* on Chr2 (peak 83 cM, CI = 77–86 cM, DBA high) found in the DBA-apoE x 129-apoE cross [4] that overlaps with a suggestive QTL (peak 74 cM, CI = 58–103 cM, DBA high) in the B6-apoE x DBA-apoE cross as well as a suggestive QTL (peak 76 cM, CI = 57–85 cM, B6 high) in the B6-apoE x 129-apoE cross.

Finally, we note that the design of crosses may have influenced the outcome of our experiments. Thus in previous crosses to generate F2 population we mated 129-apoE males with B6-apoE or DBA-apoE females, while in the current cross DBA-apoE males were mated with B6-apoE females. A parent-of-origin effect on atherosclerosis susceptibility was evident in the study by Breslow and colleagues [26], in which the authors observed striking differences in LOD scores for QTL peaks on Chr3 and Chr12 when B6-*Ldlr*-null males were mated with FVB-*Ldrl*-null females versus when FVB- *Ldrl*-null males were mated with B6-*Ldrl*-null females. Effects of sex chromosomes and mitochondrial DNA on the development of atherosclerosis have not been adequately addressed. Overall, inheritance of atherosclerosis risk may be complicated by the involvement of multiple genes, gene-by-gene interaction or different epistatic interactions dependent on genetic background and gender. Understanding the mechanisms for these phenomena will be challenging, although important.

Most QTL studies on atherosclerosis in mice have focused on lesion development at the aortic root, and multiple overlapping QTLs identified in these experiments could also help the analysis of our three crosses. Thus, the QTL on Chr7 (peak 37 cM, CI = 23–48 cM), DBA allele which is protective for B6 root atherosclerosis, co-localizes with the female-specific *Ath31* locus revealed in F2 populations of B6-apoE and *Apoe*-null on C3H/HeJ (C3H) background (C3H-apoE) [27]. It also overlaps with the *Ath3* locus determined in F2 females from an intercross of A/J and B6 mice with wild-type *Apoe* [28], and *Aorls2* found in F2 females from B6 x DBA cross with wild-type *Apoe* [29]. If the same causative variants underlie all these QTLs, the variants are likely shared by 129 and B6 but differ from DBA, A/J and C3H. However, no DNA variant with such sharing pattern was found on Chr7 in the 23–48 cM range. Indeed the haplotype comparison of the region indicates that 129 and C3H are identical by descent over the entire range, and only a few unique variants were found by Sanger sequence analysis. We note that the region spans a cluster of imprinted genes and is homologous to human Chr15 that contains the genes causing Prader-Willi and Angelman syndromes and that imprinting of some of our candidate genes, including *Atp10a*, in mice have been described [30]. Furthermore, a high-fat, high-cholesterol diet was used to provoke atherosclerosis in the cross between B6-apoE^*-*^ and C3H-apoE [31], while mice in our three crosses were maintained on normal chow, and the diet might have subtly influenced the results. A cross between 129-apoE and C3H-apoE mice could help dissect the basis for this conflict.

In summary, our study of three F2 populations generated from circular crosses of *Apoe*-null mice on three genetic backgrounds have validated three QTLs contributing to the development of atherosclerosis that are specific for particular vascular beds and suggested some candidate genes. Further analysis would shed new light on understanding the role of genetic factors in the progression of atherosclerosis.

## Supporting information

S1 Fig Image of the plaque areas in the different vascular locations. (A) Innercurve of aortic arch (aortic arch). (B) Innominate artery. (C) Left common carotid artery. (D) Subclavian artery plus upper wall.
(TIF)

S2 Fig Correlations between plaque sizes at root and plasma lipids. (A, B) Correlations of root plaque sizes with total cholesterol in F2 males (A) and females (B) of a cross between B6-apoE and DBA-apoE mice. (C, D) Correlations of root plaque sizes with HDL-cholesterol in F2 males (C) and females (D) of the cross between B6-apoE and DBA-apoE mice. (E, F) Correlations of root plaque sizes with triglycerides in F2 males (E) and females (F) of the cross between B6-apoE and DBA-apoE mice. A multivariate regression model was used to determine correlations between root plaque sizes and plasma lipids.
(TIF)

S3 Fig Correlations between plaque sizes at arch and plasma lipids. (A, B) Correlations of arch plaque sizes with total cholesterol in F2 males (A) and females (B) of a cross between B6-apoE and DBA-apoE mice. (C, D) Correlations of arch plaque sizes with HDL-cholesterol in F2 males (C) and females (D) the of cross between B6-apoE and DBA-apoE mice. (E, F) Correlations of arch plaque sizes with triglycerides in F2 males (E) and females (F) of the cross between B6-apoE and DBA-apoE mice. A multivariate regression model was used to determine correlations between arch plaque sizes and plasma lipids.
(TIF)

S4 Fig LOD curves and allelic distribution of QTLs for arch plaque lesion. (A) LOD curve for arch lesion size with sex as an additive (red line) and an interactive (magenta line) covariates in F2 mice from a cross B6-apoE x DBA-apoE. The horizontal dashed line represents a threshold for a suggestive (p = 0.63) QTL and a dotted line represents a threshold for a significant QTL (p = 0.05) in the sex-additive model. The significance thresholds for LOD scores were determined by 1000 permutations using R/qtl software. (B) LOD curve for arch lesion size with sex as an additive (red line) covariate in F2 mice from a cross B6-apoE x DBA-apoE. The horizontal dashed line represents a threshold for a suggestive (p = 0.63) QTL and a dotted line represents a threshold for a significant QTL (p = 0.05) in the sex-additive model. The significance thresholds for LOD scores were determined by 1000 permutations using R/qtl software. (C) LOD curve for arch lesion size with sex as an interactive (magenta line) covariate in F2 mice from a cross B6-apoE x DBA-apoE. The horizontal dashed line represents a threshold for a suggestive (p = 0.63) QTL and a dotted line represents a threshold for a significant QTL (p = 0.05) in the sex-interactive model. The significance thresholds for LOD scores were determined by 1000 permutations using R/qtl software.
(TIF)

S5 Fig LOD curves and allelic distribution of QTLs for root plaque size. (A) LOD curve for root lesion size with sex as an additive (red line) and an interactive (magenta line) covariates in F2 mice from a cross B6-apoE x DBA-apoE. The horizontal dashed line represents a threshold for a suggestive (p = 0.63) QTL and a dotted line represents a threshold for a significant QTL (p = 0.05) in the sex-additive model. The significance thresholds for LOD scores were determined by 1000 permutations using R/qtl software. (B) LOD curve for root lesion size with sex as an additive (red line) covariate in F2 mice from a cross B6-apoE x DBA-apoE. The horizontal dashed line represents a threshold for a suggestive (p = 0.63) QTL and a dotted line represents a threshold for a significant QTL (p = 0.05) in the sex-additive model. The significance thresholds for LOD scores were determined by 1000 permutations using R/qtl software. (C) LOD curve for root lesion size with sex as an interactive (magenta line) covariate in F2 mice from a cross B6-apoE x DBA-apoE. The horizontal dashed line represents a threshold for a suggestive (p = 0.63) QTL and a dotted line represents a threshold for a significant QTL (p = 0.05) in the sex-interactive model. The significance thresholds for LOD scores were determined by 1000 permutations using R/qtl software.
(TIF)

S1 Table QTLs for atherosclerosis at the aortic arch in F2 mice from intercross between DBA-apoE and B6-apoE mice. F, female; M, male; Chr, chromosome; CI, 95% credible interval; LOD, logarithm of odds; LOD score for sex-combined scan shown in Table was determined in single locus scan using sex as additive; for each QTL, model of inheritance was determined according to allelic effect at the nearest marker of a QTL by performing Haley-Knott regression using the additive and dominant/recessive models; ratio (*d*/*a*) was used to determine mode of inheritance [12, 13]: 0.5 <| *d/a* |< 1.5 –dominant or recessive; *d/a* = 0−pure additive; | *d/a* |≤ 0.5-additive; | *d/a* |≥1.5—overdominant or overrecessive; % variance indicates the percentage of the total F2 phenotypic variance.
(DOCX)

S2 Table QTLs for atherosclerosis at the aortic root in F2 Mice from intercross between DBA-apoE and B6-apoE mice. F, female; M, male; Chr, chromosome; CI, 95% credible interval; LOD, logarithm of odds; LOD score for sex-combined scan shown in Table was determined in single locus scan using sex as additive; for each QTL, model of inheritance was determined according to allelic effect at the nearest marker of a QTL by performing Haley-Knott regression using the additive and dominant/recessive models; ratio (*d*/*a*) was used to determine mode of inheritance [12, 13]: 0.5 <| *d/a* |< 1.5 –dominant or recessive; *d/a* = 0−pure additive; | *d/a* |≤ 0.5-additive; | *d/a* |≥1.5—overdominant or overrecessive; % variance indicates the percentage of the total F2 phenotypic variance.
(DOCX)

S3 Table Predicted effects of amino acid substitutions in candidate genes for Chr7, CI = 23-48cM (37-84Mb). Gene expression levels in the aortic arch estimated by the microarray analysis in the wild-type C57BL/6J (B6), DBA/2J (DBA) and 129S6/SvEvTac (129) strains [6] are shown. Values are mean±SE of the intensity values from three samples pooled from 5 aortic arches per strain, and statistical analysis was carried out using one-way ANOVA; Chr, chromosome; CI, 95% credible interval.
(DOCX)

S4 Table Aortic arch expression of genes near Chr 7, CI = 23-48cM (37-84Mb) that differs between B6 and DBA mice. Expression levels of the genes in the aortic arch were estimated by the microarray analyses of the wild-type C57BL/6J (B6), DBA/2J (DBA) and 129S6/SvEvTac (129) strains [6] are shown. Values are mean±SE of the intensity values from three samples pooled from 5 aortic arches per strain, and statistical analysis was carried out using one-way ANOVA; Chr, chromosome; CI, 95% credible interval.
(DOCX)
