# Supplementary figures and images for: Genetic architecture of atherosclerosis dissected by QTL analyses in three F2 intercrosses of apolipoprotein E-null mice on C57BL6/J, DBA/2J and 129S6/SvEvTac backgrounds
Source: PLoS One. 2017 Aug 24;12(8):e0182882. doi: 10.1371/journal.pone.0182882 (PMC5570285; doi:10.1371/journal.pone.0182882)

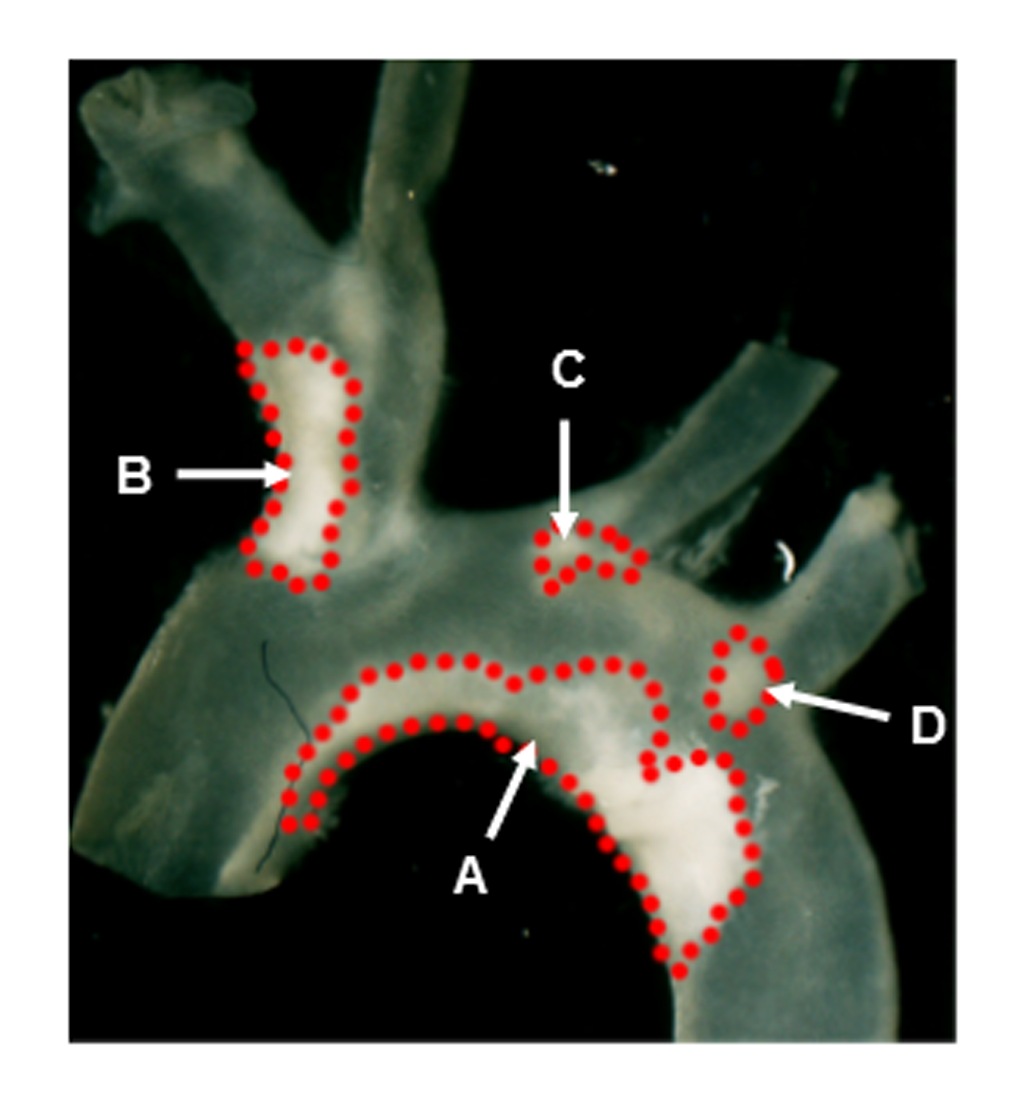

Supplement: S1 Fig — (A) Innercurve of aortic arch (aortic arch). (B) Innominate artery. (C) Left common carotid artery. (D) Subclavian artery plus upper wall. (TIF) [file pone.0182882.s001.tif]

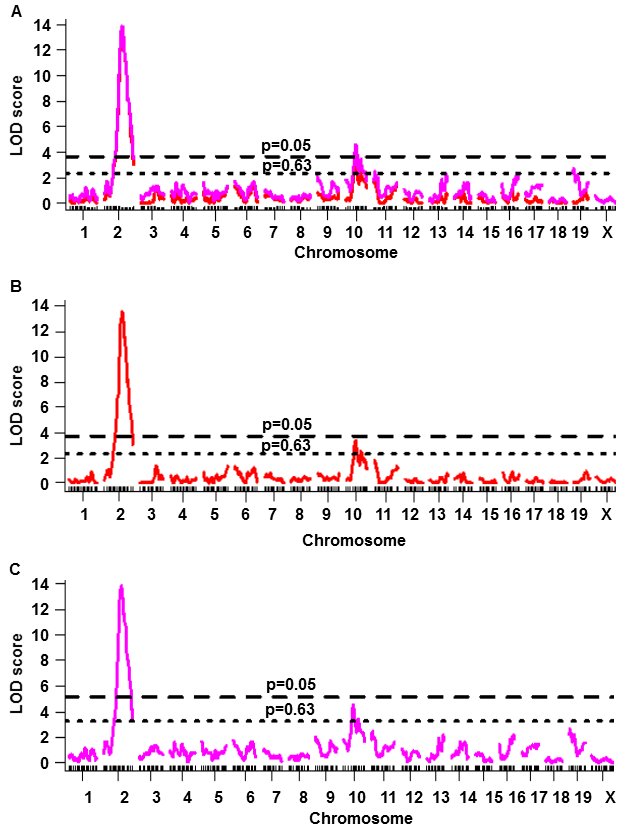

Supplement: S4 Fig — (A) LOD curve for arch lesion size with sex as an additive (red line) and an interactive (magenta line) covariates in F2 mice from a cross B6-apoE x DBA-apoE. The horizontal dashed line represents a threshold for a suggestive (p = 0.63) QTL and a dotted line represents a threshold for a significant QTL (p = 0.05) in the sex-additive model. The significance thresholds for LOD scores were determined by 1000 permutations using R/qtl software. (B) LOD curve for arch lesion size with sex as an additive (red line) covariate in F2 mice from a cross B6-apoE x DBA-apoE. The horizontal dashed line represents a threshold for a suggestive (p = 0.63) QTL and a dotted line represents a threshold for a significant QTL (p = 0.05) in the sex-additive model. The significance thresholds for LOD scores were determined by 1000 permutations using R/qtl software. (C) LOD curve for arch lesion size with sex as an interactive (magenta line) covariate in F2 mice from a cross B6-apoE x DBA-apoE. The horizontal dashed line represents a threshold for a suggestive (p = 0.63) QTL and a dotted line represents a threshold for a significant QTL (p = 0.05) in the sex-interactive model. The significance thresholds for LOD scores were determined by 1000 permutations using R/qtl software. (TIF) [file pone.0182882.s004.tif]

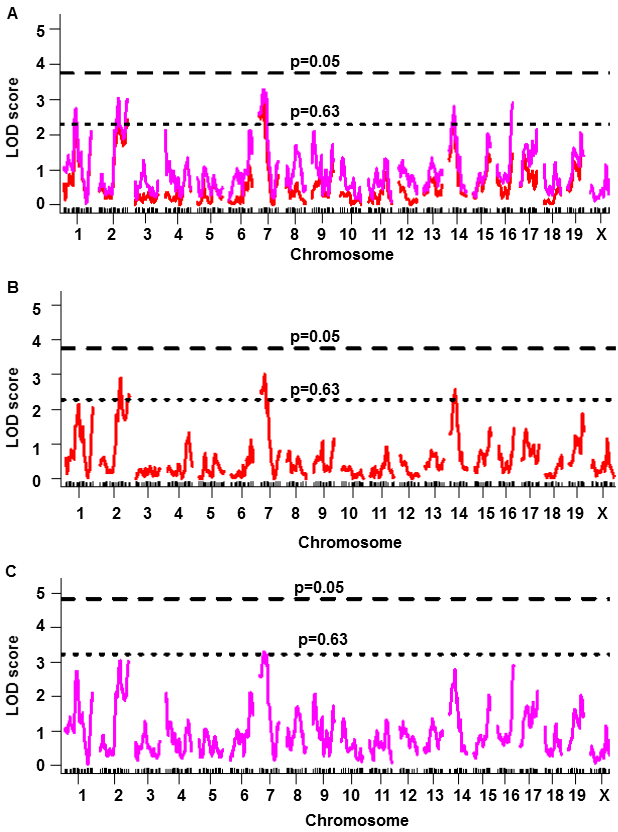

Supplement: S5 Fig — (A) LOD curve for root lesion size with sex as an additive (red line) and an interactive (magenta line) covariates in F2 mice from a cross B6-apoE x DBA-apoE. The horizontal dashed line represents a threshold for a suggestive (p = 0.63) QTL and a dotted line represents a threshold for a significant QTL (p = 0.05) in the sex-additive model. The significance thresholds for LOD scores were determined by 1000 permutations using R/qtl software. (B) LOD curve for root lesion size with sex as an additive (red line) covariate in F2 mice from a cross B6-apoE x DBA-apoE. The horizontal dashed line represents a threshold for a suggestive (p = 0.63) QTL and a dotted line represents a threshold for a significant QTL (p = 0.05) in the sex-additive model. The significance thresholds for LOD scores were determined by 1000 permutations using R/qtl software. (C) LOD curve for root lesion size with sex as an interactive (magenta line) covariate in F2 mice from a cross B6-apoE x DBA-apoE. The horizontal dashed line represents a threshold for a suggestive (p = 0.63) QTL and a dotted line represents a threshold for a significant QTL (p = 0.05) in the sex-interactive model. The significance thresholds for LOD scores were determined by 1000 permutations using R/qtl software. (TIF) [file pone.0182882.s005.tif]
