# Supplementary material for: Genetic architecture of atherosclerosis dissected by QTL analyses in three F2 intercrosses of apolipoprotein E-null mice on C57BL6/J, DBA/2J and 129S6/SvEvTac backgrounds
Source: PLoS One. 2017 Aug 24;12(8):e0182882. doi: 10.1371/journal.pone.0182882 (PMC5570285; doi:10.1371/journal.pone.0182882)
Supplement: S1 Table — F, female; M, male; Chr, chromosome; CI, 95% credible interval; LOD, logarithm of odds; LOD score for sex-combined scan shown in Table was determined in single locus scan using sex as additive; for each QTL, model of inheritance was determined according to allelic effect at the nearest marker of a QTL by performing Haley-Knott regression using the additive and dominant/recessive models; ratio (d/a) was used to determine mode of inheritance [12, 13]: 0.5 <| d/a |< 1.5 –dominant or recessive; d/a = 0−pure additive; | d/a |≤ 0.5-additive; | d/a |≥1.5—overdominant or overrecessive; % variance indicates the percentage of the total F2 phenotypic variance. (DOCX) [file pone.0182882.s006.docx]

| **S1 Table. QTLs for atherosclerosis at the aortic arch in F2 mice from intercross between DBA-apoE and B6-apoE mice.**   \|  \| Chr \| Peak  (cM) \| CI  (cM) \| \| Peak  (Mb) \| \| CI  (Mb) \| \| \| LOD \| Significance \| a \| d \| d /a \| \| High  allele \| Mode \| % \| \| --- \| --- \| --- \| --- \| --- \| --- \| --- \| --- \| --- \| --- \| --- \| --- \| --- \| --- \| --- \| --- \| --- \| --- \| --- \| \| **Arch** \|  \|  \|  \| \|  \|  \| \| \| \|  \|  \|  \|  \|  \| \|  \|  \|  \| \| F+M \| 2 \| 66 \| 60-71 \| \| 135 \| 119-143 \| \| \| \| 13.6 \| Significant \| 129 \| -37 \| -0.3 \| \| DBA \| Additive \| 21.1 \| \|  \| 10 \| 35 \| 28-69 \| \| 66 \| 55-121 \| \| \| \| 3.4 \| Suggestive \| -41 \| -79 \| 1.9 \| \| B6 \| Overecessive \| 4.0 \| \| F \| 2 \| 61 \| 58-70 \| \| 105 \| 96-128 \| \| \| \| 8.5 \| Significant \| 130 \| -63 \| -0.5 \| \| DBA \| Additive \| 25.1 \| \| M \| 2 \| 67 \| 59-80 \| \| 155 \| 135-170 \| \| \| \| 5.6 \| Significant \| 132 \| 2 \| 0.0 \| \| DBA \| Additive \| 19.2 \| \|  \| 10 \| 35 \| 29-72 \| \| 79 \| 61-124 \| \| \| \| 3.1 \| Suggestive \| -86 \| -92 \| 1.1 \| \| B6 \| Recessive \| 9.6 \| \| **Innominate artery** \| \| \| \| \| \| \| \| \|  \| \| \| \| \| \|  \| \| \| \| \| F+M \| 2 \| 60 \| 56-66 \| 119 \| \| 128-154 \| \| \| \| 12.6 \| Significant \| 105 \| 23 \| 0.2 \| \| DBA \| Additive \| 20.5 \| \|  \| 10 \| 42 \| 29 -70 \| 86 \| \| 57-122 \| \| \| \| 2.9 \| Suggestive \| -29 \| -66 \| 2.2 \| \| B6 \| Overecessive \| 3.9 \| \| F \| 2 \| 64 \| 59-70 \| 115 \| \| 100-128 \| \| \| \| 9.9 \| Significant \| 128 \| 36 \| 0.3 \| \| DBA \| Additive \| 25.8 \| \|  \| 4 \| 18 \| 10-71 \| 30 \| \| 19-127 \| \| \| \| 2.3 \| Suggestive \| 1 \| -95 \| -160 \| \| DBA \| Overrecessive \| 4.8 \| \| M \| 2 \| 58 \| 48-88 \| 132 \| \| 98-173 \| \| \| \| 4.1 \| Suggestive \| 81 \| 0 \| 0.0 \| \| DBA \| Additive \| 13.9 \| \|  \| 10 \| 36 \| 2-68 \| \| 79 \| 7-122 \| \| \| \| 2.7 \| Suggestive \| -51 \| -74 \| 1.5 \| \| B6 \| Overrecessive \| 8.4 \| \| **Left common carotid artery** \| \| \| \| \| \| \| \| \|  \| \| \| \| \| \|  \| \| \| \| \| F+M \| 1 \| 79 \| 74-83 \| \| 171 \| \| 166-177 \| \| \| 6.5 \| Significant \| 40 \| -32 \| -0.8 \| \| DBA \| Recessive \| 9.4 \| \|  \| 2 \| 63 \| 58-73 \| \| 129 \| \| 114-147 \| \| \| 6.0 \| Significant \| 40 \| 22 \| 0.6 \| \| DBA \| Dominant \| 8.2 \| \|  \| 8 \| 43 \| 9-65 \| \| 88 \| \| 17-118 \| \| \| 2.4 \| Suggestive \| 28 \| -6 \| -0.2 \| \| DBA \| Additive \| 4.3 \| \| F \| 1 \| 79 \| 76-82 \| \| 164 \| \| 156-166 \| \| \| 5.4 \| Significant \| 51 \| -33 \| -0.7 \| \| DBA \| Recessive \| 14.0 \| \|  \| 2 \| 63 \| 57-90 \| \| 108 \| \| 92-158 \| \| \| 3.7 \| Suggestive \| 43 \| 25 \| 0.6 \| \| DBA \| Dominant \| 9.0 \| \|  \| 16 \| 57 \| 50-58 \| \| 90 \| \| 76-94 \| \| \| 3.4 \| Suggestive \| 23 \| 60 \| 2.7 \| \| DBA \| Overdominant \| 6.3 \| \| M \| 1 \| 85.6 \| 61-98 \| \| 188 \| \| 159-203 \| \| \| 2.6 \| Suggestive \| 22 \| -51 \| -2.3 \| \| DBA \| Overrecessive \| 5.6 \| \|  \| 2 \| 62.6 \| 40-94 \| \| 147 \| \| 75-179 \| \| \| 2.4 \| Suggestive \| 36 \| 18 \| 0.5 \| \| DBA \| Additive \| 5.0 \| \|  \| 15 \| 44 \| 37-58 \| \| 96 \| \| 89-108 \| \| \| 2.4 \| Suggestive \| 14 \| -56 \| -4.1 \| \| DBA \| Overrecessive \| 8.1 \| \| **Subclavian artery plus upper wall** \| \| \| \| \| \| \| \| \|  \| \| \| \| \| \|  \| \| \| \| \| F+M \| 2 \| 67 \| 63-72 \| \| 136 \| \| 129-146 \| \| \| 8.7 \| Significant \| 70 \| 5 \| 0.1 \| \| DBA \| Additive \| 15.6 \| \|  \| 11 \| 83 \| 6-86 \| \| 119 \| \| \| 10-122 \| \| 3.5 \| Suggestive \| 22 \| 58 \| 2.7 \| \| DBA \| Overdominant \| 6.7 \| \| F \| 2 \| 66 \| 62-74 \| \| 116 \| \| 106-135 \| \| \| 5.5 \| Significant \| 77 \| -5 \| -0.1 \| \| DBA \| Additive \| 10.6 \| \|  \| 5 \| 24 \| 2-31 \| \| 41 \| \| 4-52 \| \| \| 2.5 \| Suggestive \| 53 \| 40 \| 0.7 \| \| DBA \| Dominant \| 3.2 \| \|  \| 19 \| 15 \| 5-24 \| \| 17 \| \| 6-25 \| \| \| 2.3 \| Suggestive \| 48 \| 10 \| 0.2 \| \| DBA \| Additive \| 5.5 \| \| M \| 2 \| 67 \| 55-81 \| \| 155 \| \| 126-170 \| \| \| 3.6 \| Suggestive \| 64 \| 14 \| 0.2 \| \| DBA \| Additive \| 14.9 \|   F, female; M, male; Chr, chromosome; CI, CI, 95% credible interval; LOD, logarithm of odds; LOD score for sex-combined scan shown in Table was determined in single locus scan using sex as additive; for each QTL, model of inheritance was determined according to allelic effect at the nearest marker of a QTL by performing Haley-Knott regression using the additive and dominant/recessive models; ratio (d/a) was used to determine mode of inheritance [11, 12]: 0.5 <│ d/a │< 1.5 –dominant or recessive;  d/a = 0 - pure additive; │ d/a │≤ 0.5-additive; │ d/a │≥1.5 - overdominant or overrecessive; % variance indicates the percentage of the total F2 phenotypic variance. |  |
| --- | --- | --- | --- | --- | --- | --- | --- | --- | --- | --- | --- | --- | --- | --- | --- | --- | --- | --- | --- | --- | --- | --- | --- | --- | --- | --- | --- | --- | --- | --- | --- | --- | --- | --- | --- | --- | --- | --- | --- | --- | --- | --- | --- | --- | --- | --- | --- | --- | --- | --- | --- | --- | --- | --- | --- | --- | --- | --- | --- | --- | --- | --- | --- | --- | --- | --- | --- | --- | --- | --- | --- | --- | --- | --- | --- | --- | --- | --- | --- | --- | --- | --- | --- | --- | --- | --- | --- | --- | --- | --- | --- | --- | --- | --- | --- | --- | --- | --- | --- | --- | --- | --- | --- | --- | --- | --- | --- | --- | --- | --- | --- | --- | --- | --- | --- | --- | --- | --- | --- | --- | --- | --- | --- | --- | --- | --- | --- | --- | --- | --- | --- | --- | --- | --- | --- | --- | --- | --- | --- | --- | --- | --- | --- | --- | --- | --- | --- | --- | --- | --- | --- | --- | --- | --- | --- | --- | --- | --- | --- | --- | --- | --- | --- | --- | --- | --- | --- | --- | --- | --- | --- | --- | --- | --- | --- | --- | --- | --- | --- | --- | --- | --- | --- | --- | --- | --- | --- | --- | --- | --- | --- | --- | --- | --- | --- | --- | --- | --- | --- | --- | --- | --- | --- | --- | --- | --- | --- | --- | --- | --- | --- | --- | --- | --- | --- | --- | --- | --- | --- | --- | --- | --- | --- | --- | --- | --- | --- | --- | --- | --- | --- | --- | --- | --- | --- | --- | --- | --- | --- | --- | --- | --- | --- | --- | --- | --- | --- | --- | --- | --- | --- | --- | --- | --- | --- | --- | --- | --- | --- | --- | --- | --- | --- | --- | --- | --- | --- | --- | --- | --- | --- | --- | --- | --- | --- | --- | --- | --- | --- | --- | --- | --- | --- | --- | --- | --- | --- | --- | --- | --- | --- | --- | --- | --- | --- | --- | --- | --- | --- | --- | --- | --- | --- | --- | --- | --- | --- | --- | --- | --- | --- | --- | --- | --- | --- | --- | --- | --- | --- | --- | --- | --- | --- | --- | --- | --- | --- | --- | --- | --- | --- | --- | --- | --- | --- | --- | --- | --- | --- | --- | --- | --- | --- | --- | --- | --- | --- | --- | --- | --- | --- | --- | --- | --- | --- | --- | --- | --- | --- | --- | --- | --- | --- | --- | --- | --- | --- | --- | --- | --- | --- | --- | --- | --- | --- | --- | --- | --- | --- | --- | --- | --- | --- | --- | --- | --- | --- | --- | --- | --- | --- | --- | --- | --- | --- | --- | --- | --- | --- | --- | --- | --- | --- | --- | --- | --- | --- | --- | --- | --- | --- | --- | --- | --- | --- | --- | --- | --- | --- | --- | --- | --- | --- | --- | --- | --- | --- | --- | --- | --- | --- | --- | --- | --- | --- | --- | --- | --- | --- | --- | --- | --- | --- | --- | --- | --- | --- | --- | --- | --- | --- | --- | --- | --- | --- | --- | --- | --- | --- | --- | --- | --- | --- | --- | --- | --- | --- | --- | --- | --- | --- | --- | --- | --- | --- | --- | --- | --- | --- | --- | --- | --- | --- | --- | --- | --- | --- | --- | --- | --- | --- | --- | --- | --- | --- | --- | --- | --- | --- | --- | --- | --- | --- | --- | --- | --- | --- | --- | --- | --- | --- | --- | --- | --- | --- | --- | --- | --- | --- | --- | --- | --- | --- | --- | --- | --- | --- | --- | --- | --- | --- | --- | --- | --- | --- | --- | --- | --- | --- | --- | --- | --- | --- | --- | --- | --- | --- | --- | --- | --- | --- | --- | --- | --- | --- | --- | --- | --- | --- | --- | --- | --- | --- | --- | --- | --- | --- | --- | --- | --- | --- | --- | --- | --- | --- | --- | --- | --- | --- | --- | --- | --- | --- | --- | --- | --- | --- | --- | --- | --- |
