# Supplementary material for: Genetic architecture of atherosclerosis dissected by QTL analyses in three F2 intercrosses of apolipoprotein E-null mice on C57BL6/J, DBA/2J and 129S6/SvEvTac backgrounds
Source: PLoS One. 2017 Aug 24;12(8):e0182882. doi: 10.1371/journal.pone.0182882 (PMC5570285; doi:10.1371/journal.pone.0182882)
Supplement: S2 Table — F, female; M, male; Chr, chromosome; CI, 95% credible interval; LOD, logarithm of odds; LOD score for sex-combined scan shown in Table was determined in single locus scan using sex as additive; for each QTL, model of inheritance was determined according to allelic effect at the nearest marker of a QTL by performing Haley-Knott regression using the additive and dominant/recessive models; ratio (d/a) was used to determine mode of inheritance [12, 13]: 0.5 <| d/a |< 1.5 –dominant or recessive; d/a = 0−pure additive; | d/a |≤ 0.5-additive; | d/a |≥1.5—overdominant or overrecessive; % variance indicates the percentage of the total F2 phenotypic variance. (DOCX) [file pone.0182882.s007.docx]

| **S2 Table**. **QTLs for atherosclerosis at the aortic root in F2 Mice from intercross between DBA-apoE and B6-apoE mice.**   \|  \| Chr \| Peak  (cM) \| CI  (cM) \| Peak  (Mb) \| CI  (Mb) \| LOD \| Significance \| a \| d \| d /a \| High  allele \| Mode \| % \| \| --- \| --- \| --- \| --- \| --- \| --- \| --- \| --- \| --- \| --- \| --- \| --- \| --- \| --- \| \| Root \| \| \| \| \| \| \| \| \| \| \| \| \| \| \| F+M \| 2 \| 74 \| 58-103 \| 149 \| 114-181 \| 2.9 \| Suggestive \| 47 \| 40 \| 0.8 \| DBA \| Dominant \| 6.8 \| \|  \| 7 \| 37 \| 23-48 \| 68 \| 37-84 \| 3.0 \| Suggestive \| -49 \| 7 \| 0.1 \| B6 \| Additive \| 5.0 \| \|  \| 14 \| 22 \| 7-32 \| 40 \| 16-61 \| 2.6 \| Suggestive \| -49 \| -31 \| 0.6 \| B6 \| Recessive \| 4.9 \| \| F \| 7 \| 42 \| 23-52 \| 71 \| 37-89 \| 2.8 \| Suggestive \| -49 \| 47 \| 1.0 \| B6 \| Dominant \| 8.0 \| \|  \| 16 \| 58 \| 3-58 \| 94 \| 56-94 \| 2.7 \| Suggestive \| 62 \| 18 \| 0.3 \| DBA \| Additive \| 7.6 \| \| M \| 2 \| 102 \| 57-103 \| 189 \| 131-193 \| 2.9 \| Suggestive \| 67 \| -4 \| 0.1 \| DBA \| Additive \| 13.4 \|   F, female; M, male; Chr, chromosome; CI, 95% credible interval; LOD, logarithm of odds; LOD score for sex-combined scan shown in Table was determined in single locus scan using sex as additive; for each QTL, model of inheritance was determined according to allelic effect at the nearest marker of a QTL by performing Haley-Knott regression using the additive and dominant/recessive models; ratio (d/a) was used to determine mode of inheritance [11, 12]: 0.5 <│ d/a │< 1.5 –dominant or recessive;  d/a = 0 - pure additive; │ d/a │≤ 0.5-additive; │ d/a │≥1.5 - overdominant or overrecessive; % variance indicates the percentage of the total F2 phenotypic variance. |  |
| --- | --- | --- | --- | --- | --- | --- | --- | --- | --- | --- | --- | --- | --- | --- | --- | --- | --- | --- | --- | --- | --- | --- | --- | --- | --- | --- | --- | --- | --- | --- | --- | --- | --- | --- | --- | --- | --- | --- | --- | --- | --- | --- | --- | --- | --- | --- | --- | --- | --- | --- | --- | --- | --- | --- | --- | --- | --- | --- | --- | --- | --- | --- | --- | --- | --- | --- | --- | --- | --- | --- | --- | --- | --- | --- | --- | --- | --- | --- | --- | --- | --- | --- | --- | --- | --- | --- | --- | --- | --- | --- | --- | --- | --- | --- | --- | --- | --- | --- | --- | --- | --- | --- | --- | --- | --- | --- | --- | --- | --- | --- | --- | --- | --- |
