# Supplementary material for: Genetic architecture of atherosclerosis dissected by QTL analyses in three F2 intercrosses of apolipoprotein E-null mice on C57BL6/J, DBA/2J and 129S6/SvEvTac backgrounds
Source: PLoS One. 2017 Aug 24;12(8):e0182882. doi: 10.1371/journal.pone.0182882 (PMC5570285; doi:10.1371/journal.pone.0182882)
Supplement: S3 Table — Gene expression levels in the aortic arch estimated by the microarray analysis in the wild-type C57BL/6J (B6), DBA/2J (DBA) and 129S6/SvEvTac (129) strains [6] are shown. Values are mean±SE of the intensity values from three samples pooled from 5 aortic arches per strain, and statistical analysis was carried out using one-way ANOVA; Chr, chromosome; CI, 95% credible interval. (DOCX) [file pone.0182882.s008.docx]

**S3 Table.** **Predicted effects of amino acid substitutions in candidate genes for Chr7, CI=23-48cM (37-84Mb).**

| Gene | Position  (Mb) | SNP | Substi-tution | Provean prediction | SIFT prediction | Aortic Arch Expression | | | | |
| --- | --- | --- | --- | --- | --- | --- | --- | --- | --- | --- |
|  |  |  |  | (cutoff=-2.5) | (cutoff=  0.05) | | B6 | DBA | 129 | P |
| *Atp10a* | 58.8 | rs32454569 | T868R | **-3.24** | 0.151 | | 789±52 | 623±32 | 769±14 | 0.0340 |
| *Magel2* | 62.4 | rs51293879 | P298S | -1.67 | **0.000** | | 22±1 | 21±1 | 24±1 | 0.2770 |
| *Peg12* | 62.5 | rs47481992 | G53R | 0.14 | **0.023** | | 26±4 | 35±4 | 31±3 | 0.3124 |
| *Trpm1* | 64.2 | rs36273674 | P530S | 1.07 | **0.000** | | 11±1 | 10±1 | 9±0 | 0.4172 |
| *Fan1* | 64.4 | rs36625877 | T157R | -1.22 | **0.027** | | 67±3 | 65±5 | 70±6 | 0.7511 |
| *Tjp1* | 65.3 | rs33906840  rs31198114 | P1219L  R143H | -1.77  -1.61 | **0.036**  **0.036** | | 931±42 | 1040±14 | 949±18 | 0.0619 |
| *Pcsk6* | 66.0 | rs16805808 | A623T | -0.72 | **0.011** | | 1006±89 | 621±51 | 491±29 | 0.0025 |
| *Lrrk1* | 66.3 | rs47717529 | A1813T | -0.55 | **0.026** | | 385±49 | 236±14 | 324±8 | 0.0337 |
| *Lins* | 66.7 | rs49373806  rs32298989 | P73L  E421K | **-3.78**  -2.2 | **0.022**  **0.000** | | 100±0 | 111±2 | 107±3 | 0.0244 |
| *Mctp2* | 72.2 | rs31107286 | A623T | -2.47 | **0.001** | | 16±1 | 19±2 | 22±5 | 0.2384 |

For each gene expression levels in the aortic arches estimated by the microarray analyses in the wild-type C57BL/6J (B6), DBA/2J (DBA) and 129S6/SvEvTac (129) strains [6] are shown. Values are mean±SE of the intensity values from three samples pooled from 5 aortic arches per strain, and statistical analysis was carried out using one-way ANOVA; Chr, chromosome; CI, 95% credible interval.
