# Supplementary material for: Genetic architecture of atherosclerosis dissected by QTL analyses in three F2 intercrosses of apolipoprotein E-null mice on C57BL6/J, DBA/2J and 129S6/SvEvTac backgrounds
Source: PLoS One. 2017 Aug 24;12(8):e0182882. doi: 10.1371/journal.pone.0182882 (PMC5570285; doi:10.1371/journal.pone.0182882)
Supplement: S4 Table — Expression levels of the genes in the aortic arch were estimated by the microarray analyses of the wild-type C57BL/6J (B6), DBA/2J (DBA) and 129S6/SvEvTac (129) strains [6] are shown. Values are mean±SE of the intensity values from three samples pooled from 5 aortic arches per strain, and statistical analysis was carried out using one-way ANOVA; Chr, chromosome; CI, 95% credible interval. (DOCX) [file pone.0182882.s009.docx]

**S4 Table. Aortic arch expression of genes near Chr 7, CI=23-48cM (37-84Mb)**

**that differs between B6 and DBA mice.**

| Gene SNPs | | Aortic Arch Expression | | | |
| --- | --- | --- | --- | --- | --- |
|  | Position (Mb) | B6 | DBA | 129 | P |
| *Klk8* | 43.8 | 108±18 | 55±2 | 88±4 | 0.0312 |
| *Klk10* | 43.8 | 282±40 | 47±3 | 321±42 | 0.0024 |
| *Shank1* | 44.3 | 68±4 | 195±17 | 104±6 | 0.0004 |
| *Snord32a* | 45.1 | 237±37 | 373±41 | 604±80 | 0.0101 |
| *Mrgpra2a* | 47.4 | 11±1 | 22±3 | 16±0 | 0.0300 |
| *Snrpn* | 60.1 | 568±30 | 1007±86 | 698±35 | 0.004 |
| *Pcsk6* | 66.0 | 1006±89 | 620±51 | 490±29 | 0.0025 |
| *Snarpa1* | 66.0 | 232±8 | 441±10 | 296±8 | 2.5E-5 |
| *Aldh1a3* | 66.4 | 86±8 | 53±5 | 44±4 | 0.0072 |
| *St8sia2* | 73.9 | 47±6 | 84±3 | 64±4 | 0.0036 |
| *Sv2b* | 75.2 | 11±1 | 24±2 | 13±1 | 0.0019 |
| *Ngrn* | 80.3 | 162±12 | 216±6 | 229±12 | 0.0070 |
| *Fah* | 84.6 | 540±28 | 624±30 | 742±18 | 0.0045 |
| *Acan* | 88.8 | 388±51 | 128±9 | 60±4 | 0.0005 |

Expression levels of the genes in the aortic arches were estimated by the microarray analyses of the wild-type C57BL/6J (B6), DBA/2J (DBA) and 129S6/SvEvTac (129) strains [6] are shown. Values are mean±SE of the intensity values from three samples pooled from 5 aortic arches per strain, and statistical analysis was carried out using one-way ANOVA; Chr, chromosome; CI, 95% credible interval.
